# Supplementary material for: Fabrication of Durable Ordered Ta2O5 Nanotube Arrays Decorated with Bi2S3 Quantum Dots
Source: Nanomaterials (Basel). 2019 Sep 20;9(10):1347. doi: 10.3390/nano9101347 (PMC6835939; doi:10.3390/nano9101347)
Supplement: Supplementary file 1 [file nanomaterials-09-01347-s001.pdf]

Supplementary Materials

Fabrication of Durable Ordered Ta_2O_5 Nanotube Arrays Decorated by Bi_2S_3 Quantum Dots

Mateusz A. Baluk[1], Marek P. Kobylański[1*], Wojciech Lisowski[2], Grzegorz Trykowski[3], Tomasz Klimczuk[4], Paweł Mazierski[1], Adriana Zaleska-Medynska[1*]

1 Department of Environmental Technology, Faculty of Chemistry, University of Gdansk, 80-308 Gdansk, Poland
2 Institute of Physical Chemistry, Polish Academy of Science, Kasprzaka 44/52, 01-224 Warsaw, Poland
3 Faculty of Chemistry, Nicolaus Copernicus University, Torun 87-100, Poland
4 Department of Solid State Physics, Faculty of Applied Physics and Mathematics, Gdansk University of Technology, 80-233 Gdansk, Poland

Table S1. Elemental composition (in at. %) in the surface layer of N_2, NH_3, H_2, and air post-deposition annealed tantalum pentoxide (Ta_2O_5) nanotube arrays (NTs). The relative contribution of Ta oxide states (in %) was evaluated after deconvolution of the Ta 4f X-ray photoelectron spectroscopy (XPS) spectra.

Sample label	Elemental composition (at. %)						Ta oxide state (%)		
	Ta	O	C	F	S	N	Ta^{5+}	Ta^0	Ta^{1+}
							26.4 eV	21.3 eV	22.3 eV
NTs_15V_5min_N_2_450°C_1h	22.44	46.00	24.32	0.15	3.10	4.00	96.84	1.44	1.72
NTs_15V_5min_Air_450°C_1h	22.32	47.75	22.78	0.38	3.48	3.29	96.97	1.23	1.80
NTs_15V_5min_NH_3_450°C_1h	21.36	47.61	22.43	0.12	2.97	5.51	96.54	1.54	1.92
NTs_15V_5min_H_2_450°C_1h	24.02	49.94	18.92	0.11	3.38	3.63	96.89	1.39	1.72

Table S2. Efficiency of the photodegradation of toluene at irradiation with an Xe lamp (1000 W).

**Sample label**	**Photodegradation efficiency (%)**					
	5 min	10 min	15 min	20 min	25 min	30 min
NTs_10V_ no_cleaned_10min_Air	64.22 ± 3.27	90.51 ± 3.27	97.47 ± 0.79	98.51 ± 0.07	98.82 ± 0.26	99.32 ± 0.33
NTs_15V_ no_cleaned_10min_Air	93.55 ± 2.38	97.83 ± 1.29	99.07 ± 0.42	99.21 ± 0.11	99.27 ± 0.02	<LOQ
NTs_20V_ no_cleaned_10min_Air	91.05 ± 3.35	96.82 ± 2.45	99.04 ± 0.96	99.10 ± 0.54	99.20 ± 0.39	99.48 ± 0.01
NTs_10V_10min_ cleaned _Air	68.38 ± 0.35	94.65 ± 1.91	98.60 ± 0.00	99.21 ± 0.02	99.37 ± 0.32	99.21 ± 0.41
NTs_15V_5min_N_2_450°C_1h	95.36 ± 0.47	98.98 ± 0.06	99.29 ± 0.11	99.68 ± 0.45	99.68 ± 0.45	99.68 ± 0.45
NTs_15V_5min_Air_450°C_1h	91.86 ± 1.22	98.73 ± 0.47	99.10 ± 0.14	99.60 ± 0.57	<LOQ	<LOQ
NTs_15V_5min_NH_3_450°C_1h	93.37 ± 2.16	97.50 ± 2.40	98.90 ± 0.67	99.21 ± 1.11	9 9.40 ± 0.85	99.51 ± 0.69
NTs_15V_5min_H_2_450°C_1h	94.20 ± 0.42	98.90 ± 0.00	99.50 ± 0.28	<LOQ	<LOQ	<LOQ
NTs_15V_5min_N_2_450°C_1h_two_step	89.28 ± 3.43	98.09 ± 0.03	99.30 ± 0.01	99.24 ± 0.01	99.39 ± 0.20	99.70 ± 0.42
NTs_15V_5min_N_2_450°C_1h_QDs_1_cycle	99.17 ± 0.14	<LOQ	<LOQ	<LOQ	<LOQ	<LOQ
NTs_15V_5min_N_2_450°C_1h_QDs_2_cycle	71.56 ± 1.12	96.53 ± 0.30	<LOQ	<LOQ	<LOQ	<LOQ
NTs_15V_5min_N_2_450°C_1h_QDs_3_cycle	inactive					

Table S3. Size of quantum dots (QDs) (based on Transmission Electron Microscopy (TEM) analysis) and elemental composition (EDX) for nanotube arrays (NTs) modified by QDs.

Sample label	Size of	Elemental composition (at. %)			
	QDs (nm)	Ta	O	Bi	S
NTs_15V_5min_N_2_450°C_1h_QDs_SILAR 1x	10.3 ± 0.9	72.69	73.36	0.30	5.5
NTs_15V_5min_N_2_450°C_1h_QDs_SILAR 2x	11.0 ± 0.8	6.26	92.69	0.32	0.71
NTs_15V_5min_N_2_450°C_1h_QDs_SILAR 3x	5.7 ±1.6	21.0	73.17	1.24	4.57

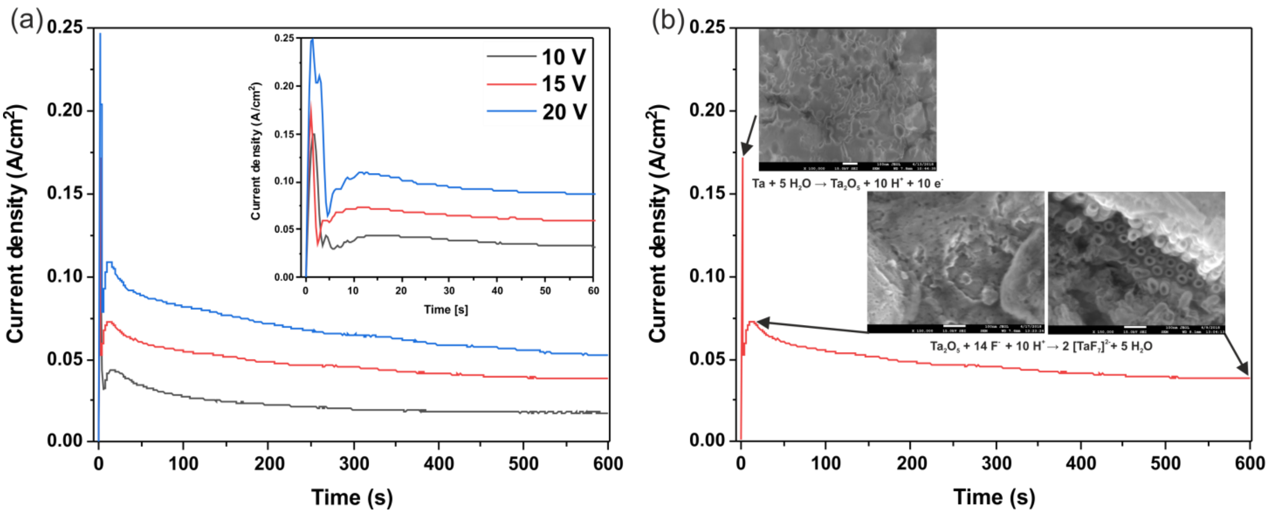

Figure S1. a) Current-density–time response for anodizing tantalum foil in the range of 10–20 V. b) The mechanism of synthesizing tantalum pentoxide (Ta_2O_5) nanotube arrays (NTs) by the anodising method (15 V, 5 min).

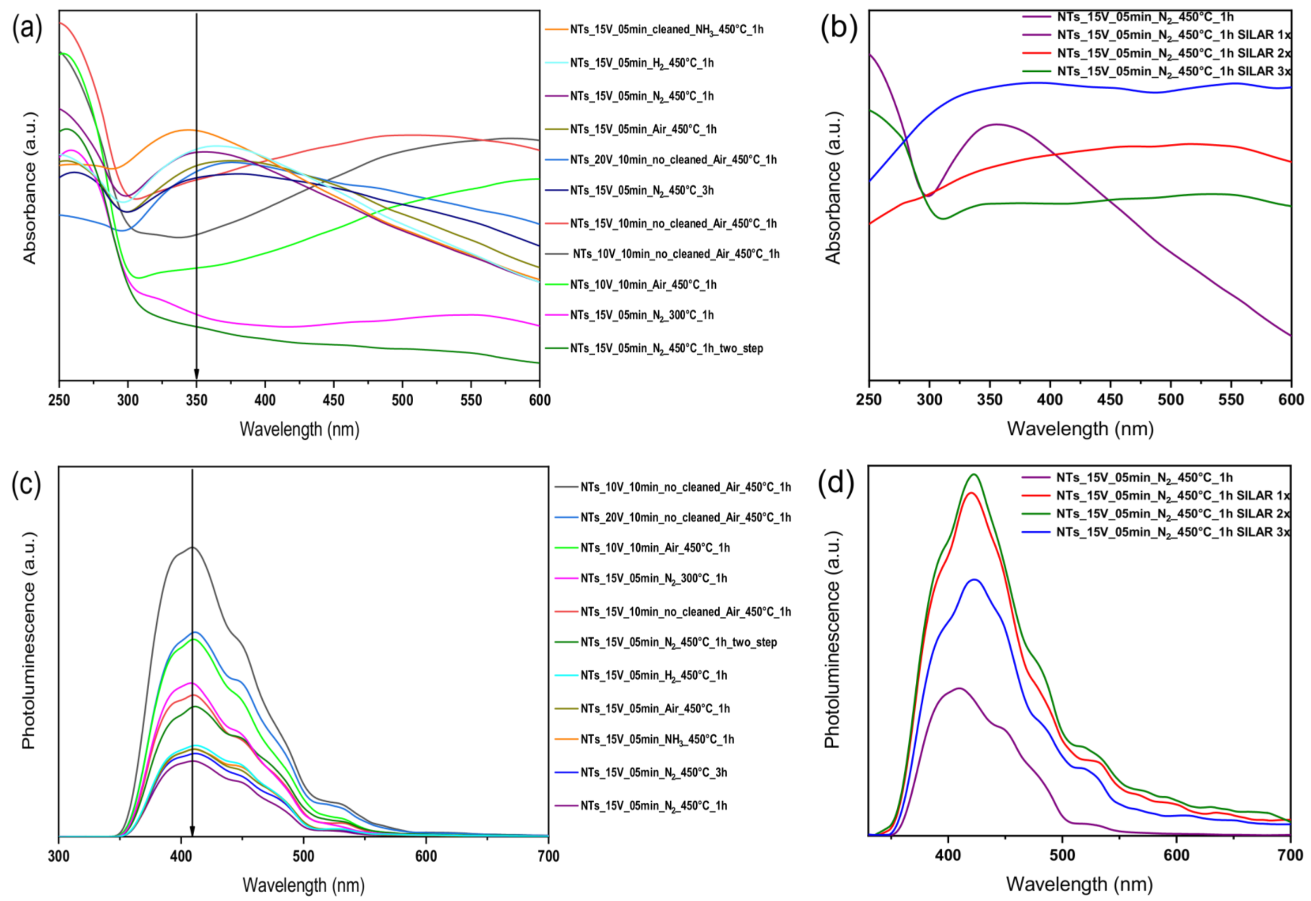

Figure S2. Optical properties: UV-Vis-DRS, photoluminescence spectra of tantalum pentoxide (Ta_2O_5) nanotube arrays (NTs) (a,b) and NT-modified bismuth sulfide (Bi_2S_3).

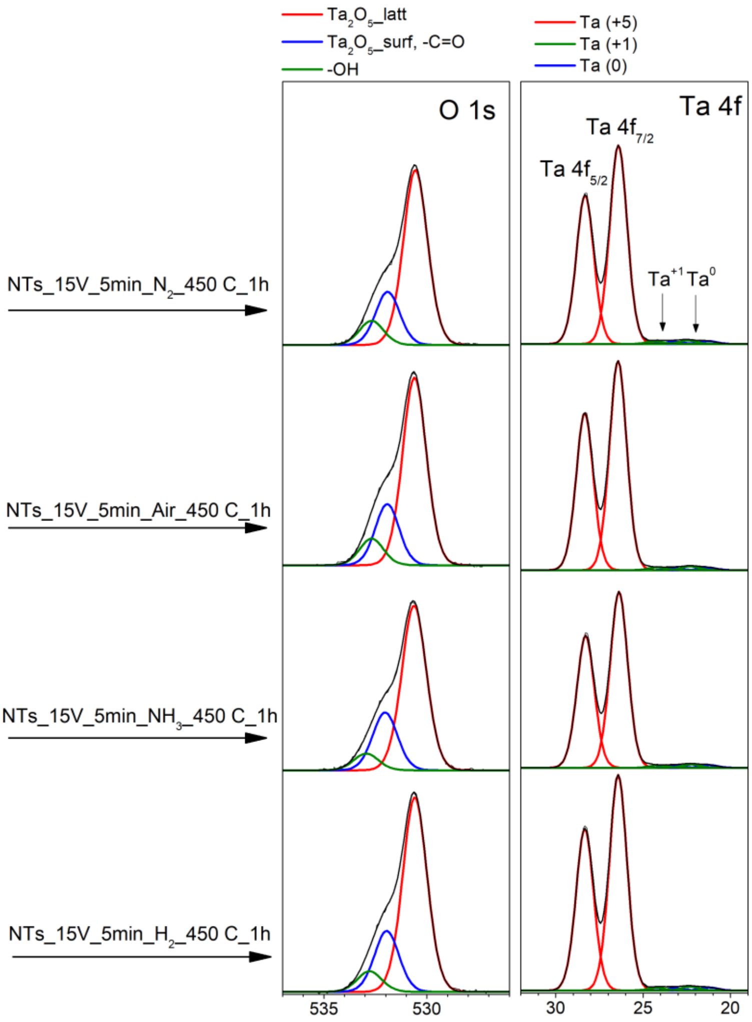

Figure S3. X-ray photoelectron spectroscopy (XPS) spectra of nanotube arrays (NTs) annealed in different atmospheres.

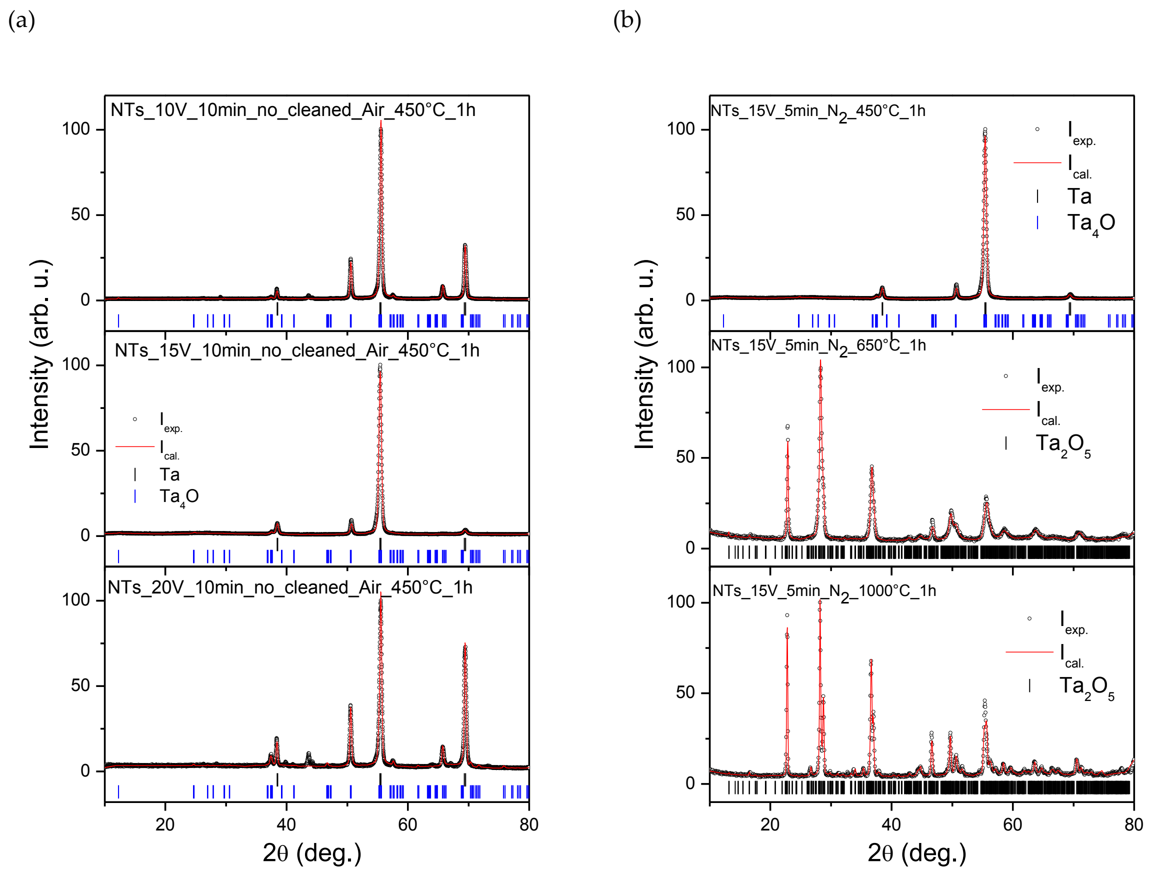

Figure S4. (a) X-ray diffraction patterns for MIB samples after the anodization process performed at 10 V, 15 V, and 20 V. (b) X-ray diffraction patterns for MIB samples calcinated at 450 ˚C, 650 ˚C and 1000 ˚C. Open circles represent experimental data, whereas the solid red line is the LeBail profile fit.

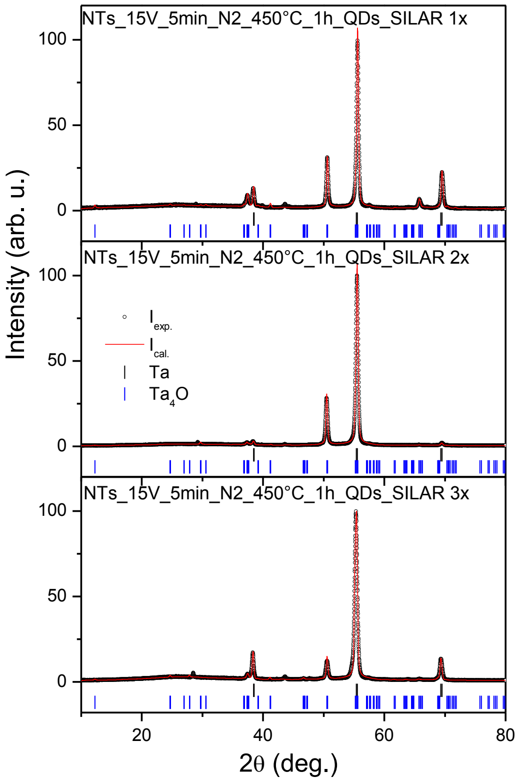

Figure S5. X-ray diffraction patterns for MIB samples after modification of bismuth sulfide (Bi_2S_3) quantum dots (QDs). Open circles represent experimental data, and the solid red line is the LeBail profile fit obtained with the two models used: Ta (Im-3m) and Ta_4O (Pmmm) shown by black and blue vertical bars, respectively.

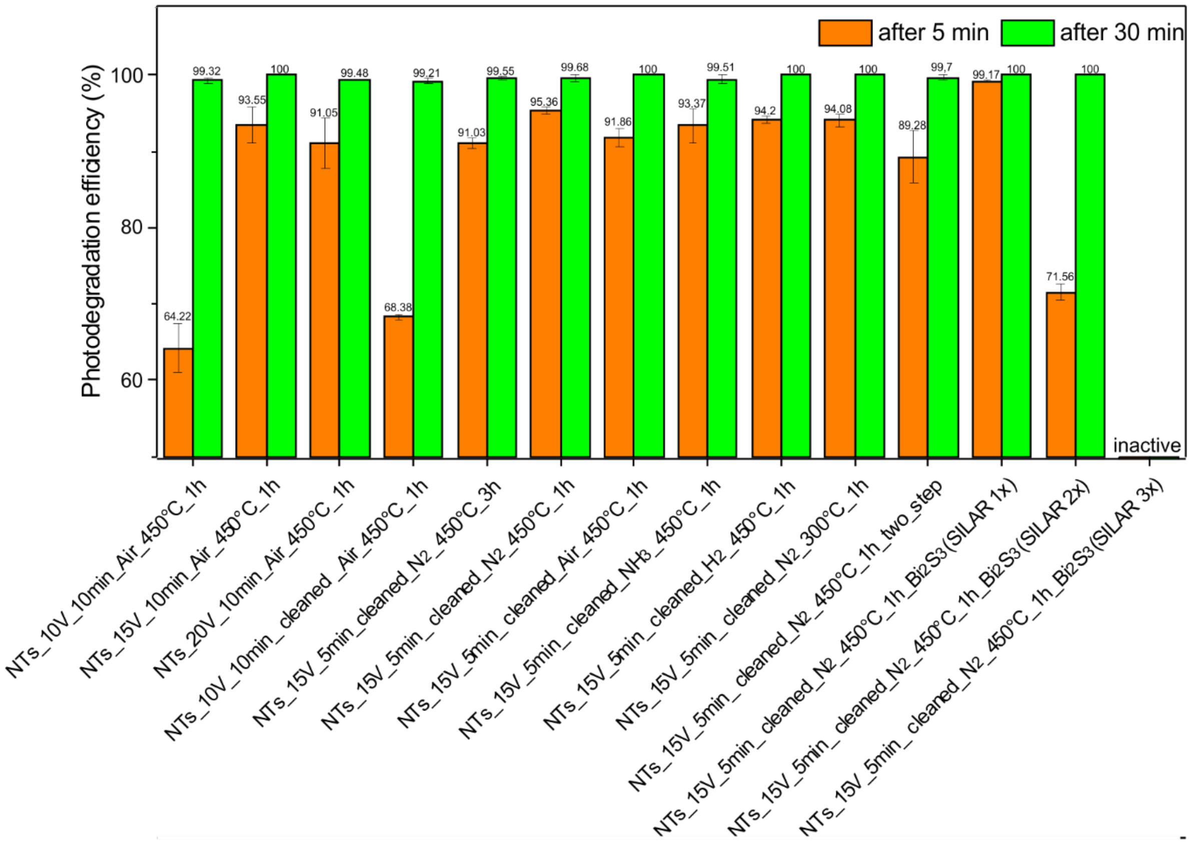

Figure S6. Photocatalytic activity of the pristine and modified tantalum pentoxide (Ta_2O_5) nanotube arrays (NTs) in toluene degradation in the gas phase (C_0 = 200 ppm) after 5 min (orange) and 30 min (green) of irradiation.

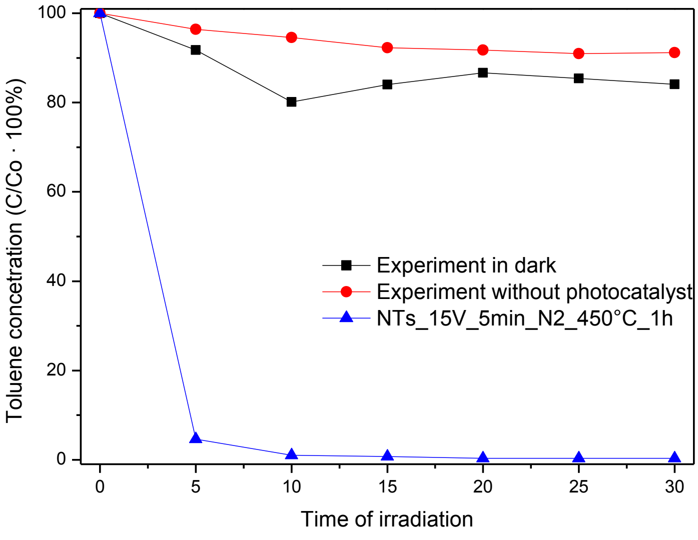

Figure S7. Efficiency of toluene removal in blank tests and during photodegradation (C_0 = 200 ppm).

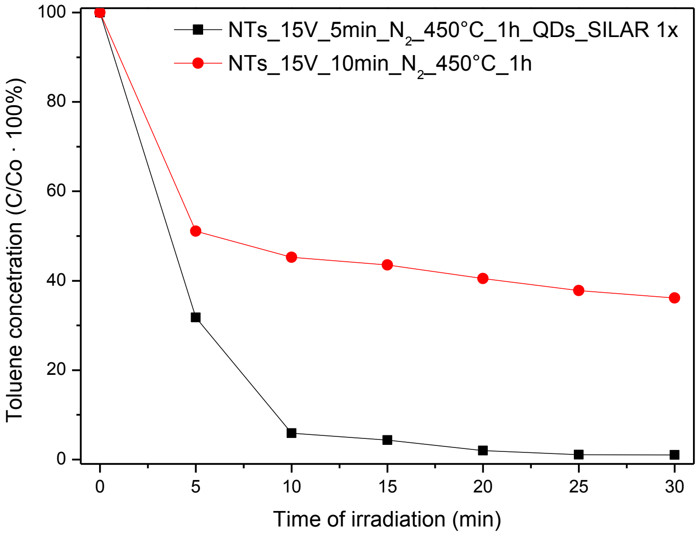

Figure S8. Kinetics of toluene degradation during irradiation over pristine tantalum pentoxide (Ta_2O_5) nanotubes and bismuth sulfide (Bi_2S_3) quantum dots (QDs)/Ta2O5 nanotube arrays (NTs) for an initial toluene concentration of 400 ppm.

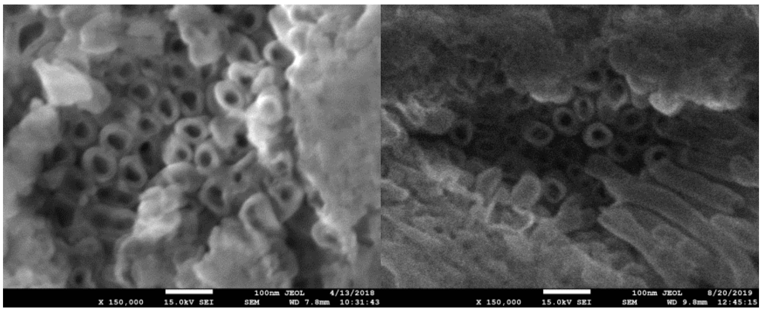

Figure S9. Surface of the samples (a) before and (b) after three cycles of photodegradation for NTs_15V_5min_N_2_450°C_1h_QDs_SILAR 1x.
